# Recent Advances in Phenolic Metabolites and Skin Cancer

**DOI:** 10.3390/ijms22189707

**Published:** 2021-09-08

**Authors:** Teodora Daria Pop, Zorita Diaconeasa

**Affiliations:** Faculty of Food Science and Technology, University of Agricultural Science and Veterinary Medicine Cluj-Napoca, Calea Mănăștur 3-5, 400372 Cluj-Napoca, Romania; Teodora-daria.pop@student.usamvcluj.ro

**Keywords:** skin cancer, melanoma, polyphenols, chemoprevention

## Abstract

Skin cancer represents any tumor development from the cutaneous structures within the epidermis, dermis or subcutaneous tissue, and is considered to be the most prevalent type of cancer. Compared to other types of cancer, skin cancer is proven to have a positive growth rate of prevalence and mortality. There are available various treatments, including chemotherapy, immunotherapy, radiotherapy and targeted therapy, but because of the multidrug resistance development, a low success has been registered. By this, the importance of studying naturally occurring compounds that are both safe and effective in the chemoprevention of skin cancer is emphasized. This review focuses on melanoma because it is the deadliest form of skin cancer, with a significantly increasing incidence in the last decades. As chemopreventive agents, we present polyphenols and their antioxidant activity, anti-inflammatory effect, their ability to balance the cell cycle and to induce apoptosis and their various other effects on skin melanoma. Besides chemoprevention, studies suggest that polyphenols can have treating abilities in some conditions. The limitations of using polyphenols are also pointed out, which are related to their poor bioavailability and stability, but as the technology is well developed, it is possible to augment the efficacy of polyphenols in the case of melanoma.

## 1. Introduction

As the burden of cancer incidence and mortality is rapidly growing worldwide, lowering the life expectancy, it is important to overcome it with new perspectives in order to develop strategies for keeping it under control. It is well known that the skin represents the organ that is the most exposed to the environmental factors, which is the reason why this review focuses its attention on skin cancer, especially melanoma, which is the deadliest form of skin cancer. A promising strategy that has been extensively studied is chemoprevention. Diet-related agents used as chemopreventive elements are of interest for scientists based on the fact that an appropriate diet and lifestyle are essential in preserving health and well-being [1]. In this way, several nutrients have gained attention in scientific researches, and, in the present writing, polyphenols are in the spotlight. The present review outlines the current discoveries according to the involvement of polyphenols in melanoma skin cancer chemoprevention and treatment.

## 2. Methods

This paper is an overview of the involvement of polyphenols in melanoma skin cancer chemoprevention and treatment.

The literature search took place in the PubMed, Web of Science, Scopus and the academic search engine Google Scholar databases. The following keywords were used: polyphenols* AND skin cancer, polyphenols* AND melanoma, polyphenols* AND squamous cell carcinoma, polyphenols* AND basal cell carcinoma and polyphenols* AND chemoprevention. The results were screened based on their titles, abstracts and full-text availability. All non-English publications were excluded from the present review. Filter limits (such as text availability, article type and publication date) were not applied. The time window was up to 1 July 2021.

## 3. Skin Cancer

The skin is the largest organ of the body and its main role is to act as a physicochemical barrier that protects internal organs against the effects of various harmful substances, which are predominantly environmental pollutants and solar ultraviolet (UV) radiation [2]. Histologically, skin has been divided into the epidermis, which is the outer layer, and dermis, and is made up of different types of cells. The epidermis contains squamous cells, basal cells and melanocytes, which are cells that provide the color of the skin. The dermis, on the other hand, contains nerves, blood vessels and sweat glands [3]. Tumor development is a multistage process characterized by the loss of cell differentiation, uncontrolled cell proliferation, invasion into the host tissue and the evasion of the host immune response [4]. Skin cancer can be divided into melanoma and non-melanoma cancer, the latter comprising mainly squamous cell carcinoma (SCC) and basal cell carcinoma (BCC) [5].

**Figure 1:**
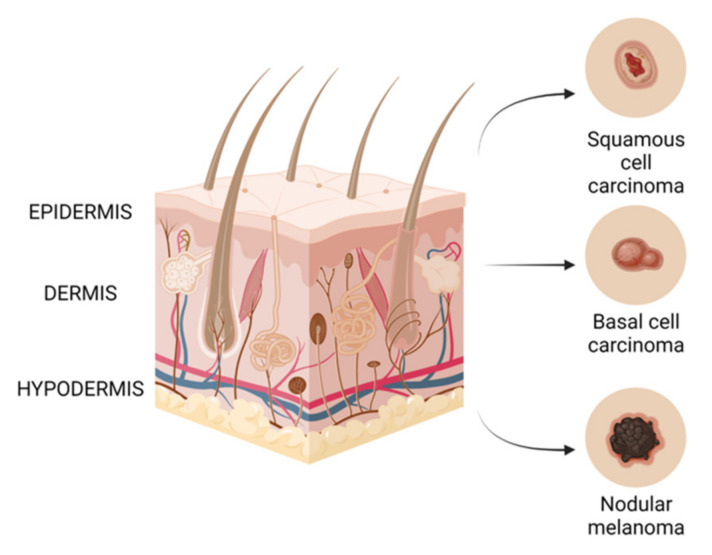
Types of skin cancer.

Non-melanomatous skin cancer (NMSC), Figure 1 is higher in incidence than melanomas, but is easier to treat, has a better long-term prognosis and is less deadly. Both basal cell carcinomas and squamous cell carcinomas are derived from epidermal cells named keratinocytes. The keratinocyte malignancy’s progress is mostly in the areas such as the face and arms that are most exposed to ultraviolet (UV) radiation [6]. Basal cell carcinoma is the most frequent malignancy in white people, with an increasing incidence in elderly men and young women. Due to hardly metastasizing to other organs, mortality is low, but basal cell carcinoma represents a burden on healthcare systems worldwide [7]. On the other hand, squamous cell carcinoma can invade other tissues, so is more likely to cause death [8], and some studies have suggested an increasing incidence, even if the rate of the increase varied from country to country [9]. For this reason, surgical excision is considered the main therapy for this kind of tumor, and other treatments, such as radiotherapy, are considered for unresectable lesions [10].

A malignant melanoma of the skin, also known as melanoma, Figure 1, is considered the deadliest form of skin cancer, whose incidence has increased significantly over the last decades, which makes preventive measures particularly urgent. Malignant melanoma is derived from epidermal melanocytes and is a metastasis-prone malignancy [6,7]. Melanoma progresses frequently on the trunk of men and the lower legs of women, but it can be found on the neck, head or elsewhere. If detected early, melanoma can be surgically extracted, but, on the other hand, there is a poor prognosis for advanced disease because of its characteristic to quickly invade and metastasize [6,11], and, if interesting mucosal surfaces are found, the prognosis of this tumor may be even worse [12]. The scientific literature has shown evidences for the fact that sun exposure causes mutations in critical genes for melanoma [13]. Ultraviolet B (UVB) radiation is considered to be the most mutagenic component of the ultraviolet spectrum and is responsible for the production of cyclobutane pyrimidine dimers (CPDs), which cause lesions that distort the DNA helix, suspending DNA replication and transcription. Indirectly, UVB can cause DNA damage by causing oxidative stress resulting from lipid peroxidation, reactive oxygen formation and nitrogen intermediates [14,15].

The probability of cancerous cell development depends on an individual’s genotypic and phenotypic characteristics, combined with the exposure to environmental risk factors. The characteristic that contributes the most to the risk of malignancy is skin color, with fair-skinned people being more likely to develop these tumors. Other important risk factors include the presence of a considerable number of moles, freckling and family history. UV radiation is an extrinsic factor that affects the skin in both direct and indirect ways, including DNA damage, oxidative stress, inflammation, immunosuppression, the depletion of the cutaneous defense system and the premature aging of the skin [16,17] Despite this, other risk factors include diet, stress, smoking, fluorescent light, hormone therapy and also tanning parlors, which are increasingly used nowadays [2].

Cancer ranks among the leading causes of morbidity worldwide. The global cancer burden using the GLOBOCAN 2020 estimates of cancer incidence and mortality produced by the International Agency for Research on Cancer shows that an estimated 19.3 million new cancer cases occurred in 2020. More than one million of new cases are represented by non-melanoma skin cancer, with approximately 63 thousand new deaths. On the other side is melanoma skin cancer, with more than 300 thousand new cases and approximately 57 thousand deaths. Overall, the burden of cancer incidence and mortality is rapidly growing worldwide, forming an important barrier to increasing life expectancy [18]. In this way, lots of scientists are focusing their work on finding new strategies for cancer prevention. Chemoprevention is one promising strategy that refers to the use of agents for the inhibition, delay or reversal of carcinogenesis before invasion. There are four major categories for cancer chemopreventive agents, including hormonal, medications, diet-related agents and vaccines. During recent years, there has been an increasing interest in the cancer chemopreventive properties of diet-derived agents. According to this, several micronutrients have gained attention in the scientific community as potential cancer-prevention agents, and, among them, there are polyphenols [19].

## 4. Polyphenols

There are some treatments for the management of metastatic and/or non-metastatic melanoma, including chemotherapy, immunotherapy, radiotherapy and targeted therapy, which are highly toxic, expensive and, in some cases, ineffective due to resistance, especially in metastatic forms. In this way, new effective therapeutic schemes should be investigated in order to deliver solutions for preventing skin cancers [20]. There is a focus on thousands of phytochemicals that have antioxidant, anti-inflammatory, anticarcinogenic, antiviral and antiallergic properties and are present in fruits and vegetables. In vivo and in vitro analyses, as well as human studies, have suggested that polyphenols have protective effects against diseases. It has also been demonstrated that the consumption of polyphenols is associated with a decreased risk of tumor development [21]. Chemopreventive agents are compounds that are able to reverse, suppress or prevent the development of cancer. As they can reveal their beneficial potential at all stages of tumor formation, polyphenols are becoming increasingly important chemopreventive agents. This review will present relevant information in order to straighten the chemoprevention effect of polyphenols [22].

Dietary polyphenols are secondary metabolites of plants, involved in the defense system against diseases and pathogenic infections, and are known as the major exogenous antioxidants [23]. The antioxidant mechanism of polyphenols is due to the phenolic group, which accepts an electron of free radicals or reactive oxygen species in order to form phenoxyl radicals, which are stable, and disrupt the free radical- or ROS-induced chain reaction in the cellular components [24].

**Figure 2:**
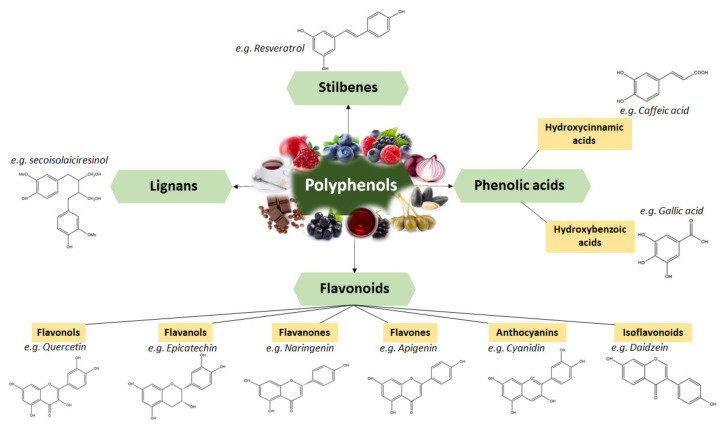
Classification and chemical structure of major classes of dietary polyphenols.

Polyphenols are classified based on their structure in several groups. Figure 2 represents the groups and subgroups of polyphenols using a representative compound for each group, showing the characteristic chemical structure and some relevant dietary sources [25].

Lignans are phenolic compounds with a 2,3-dibenzyl butane structure formed by two cinnamic acid residue dimerization [26]. Depending on the structural type and concentration, several dietary lignans have been shown to possess biological activities, and also act like antioxidants in tissues and organs [27]. As dietary sources, lignans are found in most fiber-rich plants, such as pumpkin seeds, sesame seeds and grains, including oats, wheat and barley; legumes, such as beans and soybeans; vegetables, including garlic, broccoli and carrots [28]. The lignan content of food is commonly low and generally does not exceed 2 mg/100 g. The exceptions are linseeds and sesame seeds, which contain higher amounts of lignans [29]. Linseeds are principally the richest known source of lignans (~300 mg/100 g), with seicoisolariciresinol (2,3-bis (3-methoxy-4-hydroxybenzyl) butane-1,4-diol) being the principal lignan from its composition [30].

Phenolic acids are the most predominant class of bioactive compounds present in various sources, including fruits and vegetables, spices, grains and beverages [31,32] Phenolic acids have gained attention due to their dietary health benefits and functionalities, which include antioxidant, anti-inflammatory, anti-allergic, immunoregulatory, anti-allergic and anti-cancer activity [33,34] Chemically, phenolic acids are phenolic compounds that have one carboxylic acid group. Typically, they are present in bound form, such as amides, esters or glycosides, and are rarely present in free form [35]. They are mainly divided into two sub-classes: hydroxybenzoic and hydroxycinnamic acids [36]. The latter are derived from cinnamic acid having the framework of a C_6_-C_3_ structure [37] and are often present in foods as simple esters with quinic acid or glucose. Hydroxycinnamic acids are more prevalent in nature than hydroxybenzoic acids and usually occur in conjugated forms. The most common hydroxycinnamic acids from dietary sources are ferulic, caffeic, p-coumaric and sinapic acids. Caffeic acid and quinic acid, together, form chlorogenic acid, which is found in numerous fruits and some vegetables [36]. Studies have shown that vegetables accumulate lower concentrations of hydroxycinnamic acids than fruits and beverages [37]. On the other side, hydroxybenzoic acids possess a common structure of C_6_-C_1_, are derived from benzoic acid and are found in soluble form (conjugated with sugars or organic acids) and bound with cell wall fractions [38,39]. Gallic acid, p-hydroxybenzoic acid, salicylic acid, ellagic acid, protocatechuic acid, syringic acid and vanillic acid are the major hydroxybenzoic acids, which differ from each other based on the modifications of the aromatic ring. Dietary sources generally have low levels of hydroxybenzoic acids, the exceptions being red fruits, onions and black radishes, containing up to 270 mg/kg fresh weight [40].

Stilbenes are phenolic compounds that have been recognized as phytoalexins and are associated with the defense mechanisms of plants, as they are produced after infections by pathogens or exposure to UV radiation. The stilbene structure is based on the C_6_-C_2_-C_6_ backbone, defined by two aromatic rings linked by an ethylene bridge [41]. The most common stilbene is resveratrol, which has been extensively studied due to its biological activities, including its suppression of inflammation and modulation of cell proliferation, angiogenesis and redux status [42]. One of the highest amounts of stilbenes is found in grapes. Stilbenes can be also found in berries, but at much lower concentrations [43]. Apart from berries, studies have detected others dietary sources for stilbenes, such as bananas, guavas, lychees, apples, peaches, pineapples, pears and beer [44,45,46,47,48].

Flavonoids represent one of the major classes of polyphenols, having a common structure consisting of two aromatic rings, bounded together by three carbon atoms that form an oxygenated heterocyclic structure (C_6_-C_3_-C_6_) [25]. Depending on the chemical structure, degree of oxidation and unsaturation of the linking chain, flavonoids can be classified into several subclasses, which include flavanols, flavanones, flavones, anthocyanidins, flavanonols and isoflavonoids [25]. Flavonoids can be found in plants in glycoside-bound and free aglycone forms [49]. Usually, excepting flavanols, flavonoids are found conjugated with various monomers or dimers of sugars, including glucose, galactose, xylose, arabinose or rhamnose [50]. Recently, interest in flavonoids has increased because of their health beneficial effects. Their health benefits have been associated with the flavonoids’ anti-tumor, anti-metastatic and anti-inflammatory activities. In addition, flavonoids act as signaling molecules modulating cell growth, inducing apoptosis, reducing ROS production and presenting potential alternatives for the prevention of cancer [51]. Worldwide, there is a variation in the quantity and types of flavonoids consumed and the intake of dietary flavonoids is between 50 and 400 mg/day [52]. Studies have shown that the class of polyphenols is widely spread in different foods and beverages, such as teas, but that the richest dietary sources are fruits and vegetables [53].

Since dietary polyphenols have gained attention as a potential prevention and treatment of skin cancer, both in vitro and in vivo studies have been carried out in order to show the protective effects on biochemical processes. In the following part, this review will collate and summarize the impact of polyphenols on health, specifically its implication in cancer prevention and treatment.

### 4.1. Cytotoxic Effect of Polyphenols

The cytotoxic effect is defined as the toxicity that is caused due to the action of chemotherapeutic agents on living cells [54]. There are studies that suggest that polyphenols have the ability to activate cells that have a cytotoxic effect on skin cancer cells. A study has shown the effect of green tea polyphenols (GTPs) on UVB-induced skin cancer cells. GTPs were shown to increase the number of cytotoxic T cells (CD8^+^ cells) that are tumoricidal, indicating a major pathway by which GTPs can inhibit tumor growth [55]. Another research analyzed the activity of anthocyanin and anthocyanidin extracts from blueberry fruits on B16-F10 melanoma cells. Results have shown that anthocyanidins were more cytotoxic than anthocyanins in a time- and dose-dependent manner. The two extracts were also tested on the normal tissue L929 cell line and showed the fact that a concentration of 12.5–800 µg/mL does not affect the cell viability. Furthermore, the extracts have low or no cytotoxicity on normal cells, but a highly efficient inhibitory effect in vivo on B16-F10 metastatic murine melanoma cells [56]. Chrysin, a dietary polyphenol that belongs to the class of flavonoids called flavones, is a biologically active compound analyzed for its antitumor potential on B16-F10 melanoma cells. Results have shown that chrysin suppressed melanoma tumor growth in a time-dependent manner at an average of 71% after 21 days of treatment. Moreover, chrysin treatment increased the cytotoxic activity of NK cells, cytotoxic T lymphocytes and macrophages. NK cells, a major component of the innate immunity, play an important role in tumor surveillance and tumor elimination. Chrysin’s antitumor action on the murine melanoma model was very promising, suggesting that chrysin could be a potential candidate for future use in alternative anti-melanoma treatments [57]. Resveratrol is a novel molecule with potential in various disease models, including cancer [58]. A study determined the efficacy of resveratrol treatment in vivo on melanoma-derived DM738 and DM443 cell lines. It resulted in a significant cytotoxic effect in a dose- and time-dependent manner. In addition, scientists revealed that resveratrol is selectively cytotoxic to malignant cells at a dose of 50 µM, with no cytotoxic effect in nonmalignant cell lines. As resveratrol has the potential to selectively target malignant cell growth while retaining a low toxicity profile, it can be a good candidate for further studies as a cancer therapeutic agent [59]. Elderberries have a high anthocyanins content and have been shown to possess anti-proliferative and anti-cancer effects. An anthocyanin-enriched extract (AEE) was obtained from elderberries in order to investigate its effect on metastatic B16-F10 murine melanoma cells. Results have shown that, in a concentration-dependent manner, the total lactate dehydrogenase (LDH) secretion was increased, a fact that proves that the membranes of B16-F10 cells were affected. AEE, at a 250 µg/mL dose, induced an increase in LDH of 74% after 24 h, proving the cytotoxic effect of AEE on B16-F10 murine melanoma cells [60].

As presented, studies have shown that polyphenols exert cytotoxic effect on different melanoma cell lines, representing an important step in proving their importance in terms of skin cancer.

### 4.2. Antiproliferative Effects

Cell proliferation is a biological process in which the number of cells increases over time through cell division [54]. Studies have shown that polyphenols have antiproliferative effects on tumor cells. A study showed that treating the human melanoma cell line (A375) with thermal-treated berries has an antiproliferative effect on tumor cells, while treating the normal fibroblast cell line (HFL-1) stimulated cell proliferation in a dose-dependent manner [61]. Another study using 13 polyphenolic compounds was made in order to evaluate the antiproliferative effect for a 72 h treatment on the melanoma cell line. The results showed that the greatest effect on cell growth was assigned to the flavonols myricetin and gallic acid, and the flavones tangeretin and baicalein, in a dose-dependent manner [62]. Pomegranates are known for having a great antioxidant potential because of their contents of phenolic compounds, flavonoids, anthocyanins, tannins, ascorbic acids and gallic acid. A study analyzed the antiproliferative effects of black pomegranate peel extract (PPE) on the B16-F10 melanoma cells. Results showed that black pomegranate peel extract has an antiproliferative effect in a dose-dependent manner on B16-F10 melanoma cells. It is not known which compound from the black pomegranate peel extract has the antiproliferative effect, but it can be explained by both its antioxidant activity and its polyphenols content [63,64]. Turmeric has been used for centuries in indigenous medicine, containing a polyphenolic compound named curcumin that has been reported to have anti-inflammatory, antioxidant and anticancer actions [65]. Moreover, a study was made in order to investigate the antiproliferative potential effect of curcumin on melanoma B16-F10 cell line. Results suggested that curcumin significantly inhibited B16-F10 cell proliferation and induced a decrease in cells in the G1 phase [66]. As cell proliferation is a critical point in tumor cell development, it is important to inhibit this process with agents that possess an antiproliferative effect. Polyphenols, as several studies have demonstrated, possess this ability to inhibit proliferation, which represents an important biological process in tumor development.

### 4.3. Protection from UV Radiation

Experimental and epidemiologic studies have suggested that dietary polyphenols protect the skin from the effects of UV radiation through multiple pathways [2]. Green tea polyphenols are shown to prevent the UV-induced photodamage of the skin and cancer [67]. The most common polyphenols from green tea are flavanols (-)-epigallocatechin, (-)-epigallocatechin-3-gallate, (-)-epicatechin, (-)-epicatechin gallate, (+)-catechin and (+)-gallocatechin. Due to its flavanols, studies have shown that the consumption or topical application of green tea reduces adverse effects of UV exposure, including skin damage, lipid peroxidation and erythema [68]. A topical application of green tea polyphenols before sun exposure protects through systemic immune suppression, inhibiting the UVB-induced infiltration of inflammatory leukocytes [69]. On human skin, polyphenols from green tea inhibited the UVB-induced erythema response, lowered the formation of cyclobutane pyrimidines in skin and protected the skin from oxidative stress induced by UVB [70,71]. In order to highlight the photoprotective characteristics of polyphenols, a study focused its attention on the UV absorbing characteristic of polyphenols due to their high molar extinction coefficients. In this way, the sun protection factor (SPF) for three groups of polyphenols, including stilbenes, flavonoids and some hydroxycinnamic acid homologues, was determined. From the first group analyzed, resveratrol can be taken into consideration due to its SPF value being close to 20. Regarding flavonoids, apigenin showed the best UVB protection value with a SPF = 28.8, followed by kaempferol, with a SPF = 24.9. Among hydroxycinnamic acid derivatives, the highest SPF value was registered by caffeic acid. The values presented in this study suggest that the polyphenols analyzed can be used as active components in sunscreen formulations due to their considerable UVB protection values [72].

UV radiation is considered to be one of the major factors responsible for DNA damage. There is direct damage when UV photons interact with DNA, or indirect damage, mainly through the enhanced production of reactive oxygen species that promote oxidative changes in DNA. The main DNA repair mechanisms are base excision repair (BER) and nucleotide excision repair (NER). BER is responsible for removing small lesions, such as oxidized 8-oxoG, occurring in seven steps. NER is important for the repair of DNA damage induced by UVR, such as thymine dimers and 6-4 PPs [73]. A study that investigated the influence of a green tea polyphenol treatment on human skin revealed a decrease in the formation of UVB-induced cyclobutane pyrimidine dimers (CPD’s), which are molecular lesions in the DNA via photochemical reactions. Moreover, another study in a murine photocarcinogenesis model demonstrated that green tea polyphenols reduce the risk of skin cancer by a reduction in UV-induced DNA damage, an effect mediated via interleukin (IL)-2, which was previously shown to induce DNA repair [74]. As UV-induced damage is a factor in skin cancer development, polyphenols have gained much attention, as they can be natural alternatives for melanoma prevention [75].

### 4.4. Antioxidant Effects

The skin’s antioxidant mechanisms protect it from the harmful effects of environmental factors and carcinogens, including UV radiation, which generates free radicals [76]. Still, when there is a persistent exposure to the factors mentioned above, the antioxidant activity becomes weaker, or, in some cases, inefficient, and this leads to immunosuppression and to the development of skin cancers. Extensive exposure to carcinogens leads to the overproduction of nitric oxide (NO), hydrogen peroxide (H_2_O_2_) and other reactive oxygen species (ROS) due to epidermal lipid peroxidation, which is the excessive infiltration of leukocytes into the skin, producing oxidative stress in cells. The main cause of ROS generation is metal ions, which play an important role in the generation of oxidative stress, DNA damage and cell death. Preventing the oxidative stress caused by ROS and RNS has important implications in the prevention and treatment of diseases such as skin cancer. Polyphenols protect cell constituents against oxidative damage by scavenging free radicals [2]. In this radical scavenging mechanism, polyphenols reduce ROS or RNS after generation, preventing damage to biomolecules or the formation of more reactive ROS. Iron has become a target of many antioxidant therapies because it represents the main cause of ROS generation and because of its role in oxidative stress, cell death and DNA damage. The reason why polyphenols as antioxidants have been extensively examined is due to their ability to interact with iron [77]. Lignans are complex bioactive polyphenolic compounds and, as dietary sources, lignans are found in most fiber-rich plants [28]. Studies have demonstrated a strong protective effect against several diseases and antioxidant properties. The antioxidant activity of lignans can be explained through different mechanisms, such as decreasing ROS generation, lipid peroxidation and protein and DNA oxidation, increasing the tissues’ antioxidant enzyme capacity and regulating key molecules involved in oxidative stress [27]. From the category of flavonoids, the anthocyanin content and antioxidant activity of different varieties of berries have been analyzed. Three methods have been used: CUPRAC, which measures the ability of the anthocyanins-enriched extract to reduce cupric ion (Cu^2+^), ABTS, which measures the ability to scavenge the radical ABTS^+^ and ORAC, which measures the capacity to scavenge the peroxyl radical. The antioxidant ability was compared with Trolox, a vitamin E analogue. The results were statistically significant, but compared with Trolox’s antioxidant capacity, the values were lower, which is something explained by the fact that only the anthocyanin was analyzed instead of all of the polyphenols from the berries [78]. Studies have suggested that numerous flavonoids are potent antioxidants. In vitro, there are several mechanisms that promote the antioxidant activity of flavonoids. One of the mechanisms is suppressing reactive oxygen species formation by the inhibition of enzymes or chelating trace elements involved in free radical production. Flavonoids are able to inhibit the enzymes responsible for superoxide anion production: the cyclooxygenase, lipoxygenase, NADH oxidase and mitochondrial succinoxidase, which are all involved in ROS generation. Trace metals play an important role in oxygen metabolism and some flavonoids have the ability to chelate them. Other mechanisms involve the capacity of flavonoids to scavenge reactive oxygen species and to upregulate or protect the antioxidant defense [79]. There are major redox transcriptional regulators that regulate ROS sources, including NRF2 and the AP-1 family members. In human melanoma SK-Mel-28, luteolin inhibited NRF2 target glutathione S-transferase at a certain dose, but, on the other hand, it stimulated NRF2 accumulation at a lower dose. This suggests that one compound can exhibit different effects on the same target at different concentrations, as flavonoids serve either as ROS scavengers or ROS stimulators [80]. Due to its presumed antioxidant activity due to its ability to chelate transition metal ions, scavenge free radicals and catalyze electron transport, quercetin has been extensively studied. Current existing literature shows that, depending on the tissue concentration, metabolism mode and bioavailability, quercetin can also act as a pro-oxidant, producing reactive quinone species and generating free radicals [81,82]. Based on this information, a study focused its attention on quercetin’s effects on the DB-1 human melanoma cell line, and the results showed that quercetin exerts pro-oxidant effect in melanoma. The oxidation of glutathione is involved in this effect and helped by the expression of the enzyme tyrosinase, which leads to apoptosis [83]. As it was presented, polyphenols have antioxidant and pro-oxidant effects on melanoma, and studies suggest that the cytoprotective and anticancer properties of polyphenols are due to these effects [84].

### 4.5. Anti-Inflammatory Effect

Inflammation is a process that constitutes a reactive response of the organism to tissue damage, representing an important factor contributing to degenerative pathologies [85]. Cyclooxygenase-2 (COX-2) is a rate-limiting enzyme for the generation of PG metabolites, and COX-2 expression has been associated with the pathophysiology of inflammation and cancer [86]. Studies have shown that the administration of a green tea polyphenols extract inhibits the UVB-induced expression of COX-2 and its PG metabolites [87]. It is also revealed that the topical application of green tea extract or EGCG significantly reduces the UVB-induced infiltration of inflammatory leukocytes and myeloperoxidase activity. In addition, the topical application of ECGC promotes the inhibition of the PG metabolite production, which plays an important role in inflammatory disorders and in proliferative skin disease [88]. Another study found that the topical application of human skin with a green tea extract reduced UV-induced p53 expression and the number of apoptotic keratinocytes, suggesting that the green tea extract may be suitable for an everyday photo chemopreventive agent [89]. Despite the green tea extract and EGCG, the topical application of resveratrol resulted in the significant inhibition of UVB-induced increases in the bio-fold skin thickness, hyperplastic response, leukocyte infiltration and COX-2 activity in SKH-1 hairless mouse skin [90,91]. Curcumin I and curcumin II (monodemethoxycurcumin), polyphenols found in turmeric, were tested on different melanoma cell lines, including SKMEL-28, M14 and UACC-62. In the anti-inflammatory study, curcumin II had the best anti-inflammatory activity against COX-1, whereas curcuim I exhibited the highest activity against the COX-2 enzyme [92]. Epidemiological studies show that the anti-inflammatory effect of dietary compounds is associated with the protective effect for some types of cancer. A recent study investigated the role of anti-inflammatory foods on melanoma, and results suggested that inflammatory markers are inversely associated with a high consumption of coffee, which leads to a lower concentration of the C-reactive protein, TNF receptor 2 and IL-6 [93].

Collectively with the other effects of polyphenols on melanoma, the studies presented provide evidence for the fact that chemopreventive effects are also mediated through polyphenols’ anti-inflammatory effects.

### 4.6. Cell Cycle and Apoptosis

The cell cycle regulates the process of cellular proliferation and growth, as well as cell division after DNA damage. Apoptosis is a process of programmed cell death and plays a major role in the selective inhibition of cancer [94,95] There are several studies suggesting that polyphenols have the ability to induce apoptosis in melanoma. In this way, sanggenol L, a natural flavonoid, was tested on B16 mouse melanoma cells, SK-MEL-2 and SK-MEL-28 human melanoma cells, and the results showed that the sanggenol L treatment inhibited the growth of the tested cells and produced significant morphological changes and alterations, revealing sanggenol L’s cell growth inhibitory effect. The study also showed that this flavonoid induced apoptosis in melanoma skin cancer B16 and SK-MEL-2 cells [94]. Another study on pomegranate polyphenols showed the ability to reduce the tumor incidence in a mouse skin tumorigenesis model by interfering with cell proliferation and stimulating apoptosis [96]. There are also studies that show that polyphenols of green tea reduce A373, Hs294t, Sk-Mel28 and SK-Mel 119 cell viability in a dose- and time-dependent manner [97]. A recent study focused its attention on resveratrol’s effects and the results showed a significant suppression in the growth of B16F10 and B6 [98], as well as a significant increase in apoptosis in B16F10 and A375 cells. In addition, the phosphorylation levels of mTOR and AKT were decreased (PI3K/AKT/mTOR axis is an important contributor to the regulation of proliferation, autophagy and cell death) [99]. Rugină et al. tested the antiproliferative and apoptotic potential of cyanidin-based anthocyanins on melanoma. Their study shows that the anthocyanins-enriched extract inhibited the proliferation of metastatic B16-F10 murine melanoma cells in a dose-dependent manner, and that the treatment induced apoptosis in the tested cell line [60]. Ellagic acid is a polyphenol that has been tested on three melanoma cell lines, including 1205Lu, WM852c and A375, and the results emphasize that ellagic acid inhibits the cell proliferation and increased levels of apoptosis. Moreover, ellagic acid decreases the synthesis of IL-1β and IL-8 and also decreases NF-kβ activity, suggesting that ellagic acid may exert a potential effect against melanoma [100]. There were observed morphological changes and a decreasing percentage of viable cells when using gallic acid in A375.S2 human melanoma. In a dose- and time- dependent manner, gallic acid also induces apoptosis, suggesting that this polyphenol might be an anti-carcinogenic compound [101]. Another study investigated the possible antitumor effect of caffeic acid on the SK-Mel-28 human melanoma cell line. After caffeic acid treatment, the cell viability decreased, inducing cell death by apoptosis, and there was a decreased gene expression of caspase. These results present caffeic acid as a potential compound for preventing tumor progression in human melanoma cells [102]. Quercetin is a polyphenol commonly found in nature, and it was also analyzed in order to observe its effects on A375SM and A375P human melanoma cells. In a concentration-dependent manner, quercetin decreased the viability and proliferation of A375SM and induced apoptosis, but no effect was observed on A375P cells. In vivo, a decrease in the tumor’s volume was observed, which is a result that indicates that quercetin inhibits the growth of A275SM melanoma cells through apoptosis, making this polyphenol an effective agent against melanoma [103]. Luteolin is also considered a promising anticancer agent for human melanoma. A recent study analyzed in vivo and in vitro the potential of luteolin against A375 melanoma cells. These findings show that luteolin inhibited proliferation and induced apoptosis in the tested cells by reducing the expression of MMP-2 and MMP-9 through the PI3K/AKT pathway [104]. As most chemotherapeutic drugs act through the induction of apoptosis, something to consider is that this effect can be exerted by polyphenols on melanoma cell lines, making these compounds suitable as a chemoprophylactic agent [105].

### 4.7. Autophagy

Autophagy’s principal role is to maintain the survival mechanism for the cell, enabling the recycling of building blocks and metabolic structures, supporting the continuous growth and the adaptive metabolic demands upon stress conditions. Due to this, autophagy has become a new potential candidate in the improved treatment of melanoma [106]. Studies have demonstrated that autophagy plays a dual role in cancer. It can have a cytoprotective function in cancer under some conditions, and may also exert anticancer effects by increasing the cytotoxicity of the chemotherapeutic agents. It is also pointed out that the autophagy depends on the genetic context, tumor type, tumor stage, microenvironment and the treatment applied. Numerous in vitro studies indicated that resveratrol is able to induce autophagy, inhibiting the Akt/mTOR pathway in B16 melanoma cells [107]. It was also shown that resveratrol suppressed the growth of B16F10 murine melanoma cells and A375 human cells by promoting autophagy and inhibiting the PI3K/AKT/mTOR signaling pathway [99]. Another study, focusing its attention on curcumin, demonstrated that the AKT/mTOR signaling pathway was downregulated, inducing autophagy in the case of A375 and C8161 human melanoma [108]. Chiu et al., in 2015, analyzed the hibiscus leaf polyphenolic extract’s potential as an antimelanoma agent. The extract was exhibited to be rich in epicatechin gallate and other polyphenols, and the results showed that the treatment induced the caspase cleavage, Bcl-2 family proteins regulation and Fas/FasL activation in the A375 melanoma cell line. Hibiscus leaf polyphenolic extract could increase the expression and autophagy, inducing autophagic cell death in A375 [109]. Prieto et al., in 2020, studied the effect of autophagy on the immunogenic signals in melanoma cells. They tested a polyphenol-rich extract from Caesalpinia spinosa in the B16F10 melanoma model, concluding that the autophagy induced by the extract delays cell death, allowing for an increase in immunogenic signals. In this way, the mechanisms used by the natural plant extract to induce immunogenic cell death can be explained, and the writers proposed a synergism between the plan extract compounds; each one even has a different action mechanism [110]. Rosenfeldt et al., 2021, examined the role of autophagy in melanoma using a mouse model containing an allele of the Braf^V600E^ mutation, the signature molecular driver of human melanoma, preceded by a Lox-STOP-Lox cassette. The exemplars were crossed to animals bearing a floxed allele of Pten, which can accelerate the disease. The findings sustain the idea that autophagy is dispensable for melanoma growth and might support a barrier function for melanoma development that, when hemizygous for Pten animals, is compromised [111]. There are also studies demonstrating that, in some conditions, blocking autophagy has advantages in the treatment of melanoma, but more studies need to be conducted in order to fully understand autophagy’s involvement in melanoma [106].

### 4.8. Tumor Metastasis

Melanoma is considered the most aggressive form of skin cancer due to its progression toward metastasis [112]. There are several steps is tumor metastasis, including cell migration, adhesion, invasion and angiogenesis [113]. The degradation of the extracellular matrix (ECM) and basement membranes are crucial steps for tumor invasion and metastasis. Tumor angiogenesis also plays a key role in melanoma metastasis and progression [114]. In order to sustain the idea that polyphenols have an inhibitory action in melanoma cancer metastasis, a study investigated the activity of anthocyanins from *Hibiscus sabdariffa* (HAs) on metastasis. Results showed that HAs significantly decreased migration in vitro and in vivo and inhibited tumor Ras, NF-kB and CD31, as well as inhibited VEGF/VEGF-R-induced angiogenesis. Overall, HAs inhibit the progression of cancer cells through the following mechanisms: the repression of migration, reduced matrix degradation and suppression of angiogenesis [115]. Another study tested several polyphenolic compounds for the inhibition of lung metastasis induced by B16F10 melanoma cells in mice. The results showed that the oral administration of curcumin and catechin at certain concentrations inhibited the lung metastases by reducing the number of nodules (80%). Other polyphenols that had the same action were found to be rutin, epicatechin, naringin and naringenin [116]. Resveratrol was also tested for its effect on three melanoma cell lines, including B16F10, B6 and A375, with lung metastasis. The lung tumor growth was inhibited in vitro using 40 mg/kg resveratrol, which is something that can be added to clinical therapeutic schemes with no additional side effects or toxicity [98].

There is a rich body of evidence suggesting that polyphenols have effects on melanoma due to their multiple mechanisms presented in the previous part. Table 1 shows in vitro and in vivo findings regarding polyphenols’ effects on melanoma.

## 5. Polyphenols’ Limitations

Although polyphenols from plants were shown to exhibit considerable effects on different melanoma cell lines, the discrepancy in effectiveness of these compounds in vitro/in vivo and in clinical applications represents a concern on their utility. Despite their potential, developing polyphenols as chemoprevention agents is limited because of various challenges, such as an ineffective systemic delivery, stability and low oral bioavailability.

Humans consume many plants and herbs that are known to contain relevant amounts of polyphenols, which are demonstrated to have a beneficial effect on health, but the levels of these compounds are influenced by intrinsic and extrinsic factors, such as genetic factors, environmental conditions, processing and storing [152]. It is important to know how much of a nutrient is present in a specific food or dietary supplement, but is even more important to how much of that is bioavailable. Bioavailability refers to the proportion of the nutrient that is digested, absorbed and metabolized through normal pathways. When studying the bioavailability, the focus is on which polyphenols are absorbed better, which polyphenols lead to the formation of the active metabolites and what active metabolites are formed [153]. For a proper study of the polyphenolic digestive fate, further studies, including the development of analytical platforms, are needed. The lack in information limits the potential of maximizing the health-promoting potential of polyphenol-rich foods [154]. Moreover, Castello et al., 2018, presented significant interindividual variability in the polyphenol metabolite profile, taking into consideration both urinary and plasma samples [155]. Despite urinary and plasma samples, there are studies that also explored the fecal excretion of metabolites and microbial-derived catabolites in order to highlight the bioavailability of polyphenols [156].

**Figure 3:**
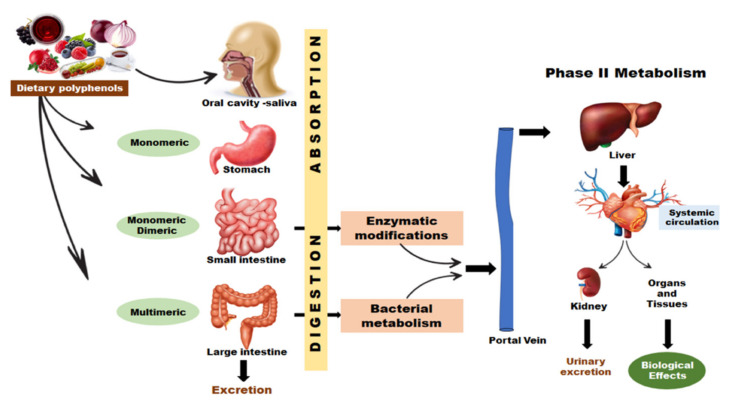
Schematic representation of polyphenols’ track when ingested. When ingested, the salivary enzymes from oral cavity exert partial hydrolysis and, then, the compounds reach stomach and small intestine. As polyphenols have limited absorption in these sections, the unabsorbed ones continue their transit to colon where they are metabolized. The metabolites are subjected to phase II metabolism and enter the bloodstream to exert biological effects. The unabsorbed polyphenols and metabolites are excreted as feces and the absorbed metabolites are mostly found in the urinary excretion.

When consumed, polyphenolic substances undergo important degradation processes due to the gastrointestinal transit, where the compounds need to be released from the food matrix in order to be absorbable. The penetration through the intestinal barrier represents a critical point due to the low amounts of bioactive compounds released into the bloodstream. The unaffected compounds undergo complex metabolism, both in the gut and the liver [157]. Figure 3 illustrates a simplified scheme that represents the polyphenols’ track when ingested.

Despite their low bioavailability, polyphenols are susceptible to various factors, such as temperature, pH and light, leading to their degradation, and, as a consequence, the reduction of their bioactivity. Once ingested, bioavailability is severely affected by both the enzyme activity and physico-chemical transformation, which is the greatest barrier to the therapeutic use of these compounds. In order to protect their bioactivity and bioavailability and to improve their release in order to obtain specific health effects, studies present encapsulation as an alternative for polyphenols’ deficiencies. Moreover, this technique increases their water solubility, thus improving their absorption rate [157]. In this way, the combination between polyphenols’ biological activity and the technological performance of nanocarriers can overcome the limitations of conventional chemotherapy and could represent an effective clinical potential for melanoma [158]. Over the years, the industry has developed several techniques in order to obtain effective delivery systems, such as phytosomes, liposomes, niosomes, protein-based nanoparticles, polymer nanoparticles, microspheres and emulsions [159]. A recent study was conducted in order to improve resveratrol’s bioavailability, as it plays a promising role in cancer prevention. In this way, a thermosensitive copolymeric nanoparticle was synthesized and was evaluated in Swiss albino mice. The best results were registered against the B16 melanoma cell line, showing a reduction in skin edema, hyperplasia and oxidative stress response. Moreover, a significant reduction was found in tumor incidence and tumor burden in the promotion phase, altering Bax and Bcl2 expressions and leading to apoptosis [58]. Using this, it can be pointed out that polyphenols’ limitations can be overcome by several technologies, enhancing their potential as cancer chemopreventive agents.

Focusing on melanoma, which is a cutaneous malignancy, we have to take into consideration other ways of delivering polyphenols than simply orally. Topical and transdermal ways of delivery have numerous advantages, such as avoiding first-pass metabolism, being a noninvasive way that facilitates the delivery of small and lipophilic compounds. In this way, a direct and localized access would be more promising for diseases such as melanoma. For efficient dermal delivery, the complex multilamellar structure of the skin should be taken into consideration, and, in this way, numerous penetration enhancement techniques have been investigated. Different nanoscale-based structures have been studied and proposed in order to increase the thermodynamic activity of the compounds and to enhance the skin penetration process [160].

## 6. Conclusions and Future Perspectives

There is a rich body of evidence highlighting the fact that polyphenols are melanoma-preventing and therapeutic potential natural agents, due to exceeding multiple mechanisms on different cell lines, both in vitro and in vivo. This review has brought together relevant studies presenting polyphenols’ influence in various melanoma cell lines, including the cytotoxic, antiproliferative, proapoptotic, anti-inflammatory and antioxidant potential. Moreover, it has been shown that these dietary compounds have the ability to balance the cell cycle, to reduce the capacity of tumor cells to metastasize and to modulate autophagy. In this line, unraveling the anticancer molecular mechanisms of these biological compounds brings an important advantage in defeating carcinogenesis and metastasis in the case of melanoma.

Taking into consideration both the efficacy and limitations of polyphenols, it can be stated that using them as therapeutic agents might overcome the poor prognosis in the case of melanoma, having a considerable impact on the management of the deadliest form of skin cancer.

As a dietary source is controversial, due to the difficulty in quantitatively establish the health benefits, it is important for the future to focus on the evidence from clinical studies that is aimed to find the most suitable way of delivering polyphenols in order to benefit from their full potential, in the case of melanoma. Nanotechnology is widely acknowledged as a potential future avenue in cancer therapy, the combination between phytochemicals and nanotechnology being a viable strategy [161].

Moreover, due to their ability to modulate the immunosuppressive effect of sun exposure, plant polyphenols represent an important area of research as additives in sunscreens.

## Figures and Tables

**Table 1:** Polyphenols’ effects on melanoma.

Flavonoids						
Subclass of Polyphenols	Compound	Sources	Study Type	Effect	Cell Lines	Ref
**Anthocyanins/** **Anthocyanidins**	Cyanidin (Cyanidin-3-o glucoside)	Red to blue fruits	In vitro	↑ apoptosis	B16-F10	[117,118]
			In vivo	↓ tumor growth	SK-MEL 1 B16-F10	
				↓ tumor growth ↓ tumor volume	B16-F10 B16-F10 luc	
	Anthocyanins enrich extract (delphinidin, cyanidin, petunidin, peonidin and malvidin)	Berries	In vitro	↓ cell growth ↓ cell proliferation ↓ viability ↑ apoptosis ↑ oxidative damage ↓ mitochondrial membrane potential	B16-F10	[56,61]
**Flavanols**	Catechin ((-)-epigallocatechin-3-gallate (EGCG))	Green tea	In vitro	↓ cell growth ↓ migration ↓ invasion	SK-MEL 5 SK-MEL 28 A375 G361	[119]
			In vivo	↓ size and number of metastatic nodules	Lung metastasis mouse B16-F10	
			In vitro	↓ cell viability ↓ cell proliferation ↑ apoptosis	A375 Hs-294T	[120]
	Theaflavin	Tea	In vitro	↓ cell proliferation ↑ apoptosis	A375	[121]
			In vivo	↓ tumor growth	A375	
**Flavanones**	Hesperetin	Citrus	In vitro	↑ melanogenesis	B16-F10	[122]
	Hesperidin	Citrus	In vitro	↑ melanogenesis	B16	[123]
**Flavones**	Apigenin	Dried parsley, celery seeds, vine-spinach and dried oregano	In vitro	↓ propagation of cells ↓ cell cycle at G2/M phase ↑ apoptosis	A375 A2058 RPMI-7951	[124,125]
			In vivo	↓ tumor growth	B16-F10	
			In vitro	↓ cell viability ↓ cell migration ↑ apoptosis ↓ tumor growth ↓ proliferation	A375P A375SM	[126]
			In vivo	↓ tumor volume ↑ apoptosis	A375SM	
			In vitro	↓ cell migration ↓ cell invasion ↑apoptosis	A375 C8161	[127]
			In vivo	↓ proliferation ↓ cell migration ↓ tumor growth	SK-MEL 24	[128]
	Baicalein and Baicalin	Edible medicinal plants	In vitro	↓ tumor growth ↓ proliferation ↑ apoptosis ↑ senescence	Mel586 SK-MEL 2 A375 B16-F0	[129]
			In vivo	↓ tumor growth ↓ tumor size ↑ apoptosis ↓ proliferation	B16F0	
	Chrysin	Honey, plants and propolis	In vitro	↓ cell proliferation ↑ cells differentiation ↑ cell death	B16-F1 A375	[130]
			In vitro	↓ cell migration ↓ metastasis	A375.S2	[131]
	Diosmin	Citrus	In vivo	↓ number of metastatic nodules ↓ growth ↓ invasion ↓proliferation	B16-F10	[132,133]
			In vivo	↓pulmonary metastasis	B16-F10	[134]
	Luteolin	Fruits, vegetables and herbs	In vitro	↓ proliferation ↓ migration ↓invasion ↑apoptosis	A375	[104]
			In vivo	↓tumor growth		
	Kaempferol	Green leafy vegetables (spinach, kale), herbs (dill, chives, tarragon)	In vitro	↓proliferation ↑apoptosis ↓cell migratory potential ↓G2/M cell cycle	A375	[135,136]
**Flavonols**	Quercetin	Onion, asparagus, berries	In vitro	↓viability ↓proliferation ↓proportion of the cell in the S and G2/M phase of cell cycle ↑apoptosis	B16	[135,137]
			In vitro	↓ invasion ↓ mobility ↓ proliferation ↓ cell rate in S and G2/M phase of cell cycle ↑ apoptosis	B16-BL6	[138,139]
			In vivo	↓ lung metastasis		
			In vitro	↓ migration ↓ invasion	A2058 A375	[140]
**Isoflavonoids**	Daidzein	Soybean	In vitro	↓growth	K1735M2	[141]
**Phenolic** **Acids**						
**Hydroxycinnamic** **Acids**	Caffeic acid	Coffee, apple, apricots, pears and berries	In vitro	↓ cell viability ↑ apoptosis ↓ colony formation	SK-Mel28	[102]
	Caffeic acid phenethyl ester (CAPE)	Propolis	In vitro In vivo	↓ growth ↑ apoptosis	SK-Mel28 B16-F0	[142]
	Cinnamic acid	Coffee, apples, citric fruits, vegetable oils and nuts	In vitro	↓ proliferation	HT-144	[143]
**Hydroxybenzoic** **Acids**	Gallic acid	Berries, plump, grapes, hazelnut, tea and wine	In vitro	↓ cell viability ↑ apoptosis ↓ mitochondrial potential↓ invasion ↓metastasis	A375.S2	[101,144,145]
			In vitro	↑ apoptosis	B16-F10	[146]
	Ellagic acid	Berries, pomegranates and nuts	In vitro	↓ growth ↑ apoptosis ↓ cell proliferation in G1 phase	1205LU WM852c A375	[100]
**Lignans**						
**Lignans**	Secoisolariciresinol diglycoside	Flaxseed	In vivo	↓ pulmonary metastasis ↓ growth of metastatic tumor	B16-BL6	[147]
**Stilbenes**						
**Stilbenes**	Resveratrol	Grapes and red wine	In vitro	↓ cell proliferation ↑apoptosis	MV3 A375	[148]
			In vitro	↓ cell viability ↑ apoptosis ↑ autophagy ↓ migration ↓ invasion	B16-F10 A375	[99]
			In vitro	↓ migration ↓ invasion ↓ tumor volume	B16-F10 B16-BL6	[149]
			In vitro	↑ apoptosis	A375 SK-Mel28	[150]
	Trans-resveratrol		In vitro	↓ colony formation ↓ cell growth ↑ cytotoxicity	M14 SK-Mel28	[151]

## Data Availability

Not applicable.
